# The Impact of COVID-19: Perspectives of Recreational Therapists Working with Older Adults

**DOI:** 10.1093/geroni/igab046.2711

**Published:** 2021-12-17

**Authors:** Betsy Kemeny, Dawn DeVries

**Affiliations:** 1 Slippery Rock University, Grove City, Pennsylvania, United States; 2 Grand Valley State University, Grand Rapids, Michigan, United States

## Abstract

This study explored the perspectives of recreational therapists (RT) from Pennsylvania and Michigan and how COVID-19 has impacted older adults and their roles in various settings. COVID-19 safety restrictions limiting social interaction with both peers and families had the potential for negatively impacting the social and emotional well-being of older adults (Van Orden et al., 2020) and the roles of therapists who work with them. Because peer socialization and physical activity programs prevent falls (Cameron et al., 2018) and improve depressive symptoms (Harvey et al., 2015), a better understanding of COVID-19 impact is significant. From a qualitative viewpoint, 14 RTs from various levels of care were interviewed to better understand their perspective on the impacts of COVID on older adults and their own practice. Hour long interviews conducted via zoom focused on organizational changes, role changes, and impact on older adults. After recordings were transcribed, multiple researchers classified, summarized, and tabulated the data. A consensus method determined common themes. From the RT’s perspective, older adults have not only been negatively impacted in the social domain. While many older adults showed resilience, others were impacted physically, emotionally, and cognitively. Moreover, an increased importance on meaningful engagement, recreation, and leisure emerged. Technology became an essential tool in interpersonal connection. Teamwork, personal self-care, and coping were integral to providing effective care. Post pandemic, RTs are concerned about the challenge of reengaging older adults in groups but are certain that technology will continue to be used in a more expansive way in programs.

